# Relationship between Malondialdehyde Serum Levels and Disease Features in a Full Characterized Series of 284 Patients with Systemic Lupus Erythematosus

**DOI:** 10.3390/antiox12081535

**Published:** 2023-07-31

**Authors:** Nayra Merino de Paz, María García-González, Fuensanta Gómez-Bernal, Juan C. Quevedo-Abeledo, Antonia de Vera-González, Raquel López-Mejias, Pedro Abreu-González, Candelaria Martín-González, Miguel Á. González-Gay, Iván Ferraz-Amaro

**Affiliations:** 1 Division of Dermatology, Dermamedicin Clínicas, 38004 Santa Cruz de Tenerife, Spain; nayradepaz@hotmail.com; 2 Division of Rheumatology, Hospital Universitario de Canarias, 38320 Santa Cruz de Tenerife, Spain; margagon23@hotmail.com; 3 Division of Central Laboratory, Hospital Universitario de Canarias, 38320 Santa Cruz de Tenerife, Spain; fuensanta95@gmail.com (F.G.-B.); adeverag@gmail.com (A.d.V.-G.); 4 Division of Rheumatology, Hospital Doctor Negrín, 35010 Las Palmas de Gran Canaria, Spain; quevedojcarlos@yahoo.es; 5 Epidemiology, Genetics and Atherosclerosis Research Group on Systemic Inflammatory Diseases, IDIVAL, 39011 Santander, Spain; rlopezmejias78@gmail.com; 6 Unit of Physiology, Department of Basic Medical Sciences, University of La Laguna, 38200 Santa Cruz de Tenerife, Spain; pabreu@ull.edu.es; 7 Division of Internal Medicine, Hospital Universitario de Canarias, 38320 Santa Cruz de Tenerife, Spain; mmartgon@ull.edu.es; 8 Department of Internal Medicine, University of La Laguna (ULL), 38200 Santa Cruz de Tenerife, Spain; 9 Division of Rheumatology, IIS-Fundación Jiménez Díaz, 28040 Madrid, Spain; 10 Department of Medicine, University of Cantabria, 39005 Santander, Spain; 11 Cardiovascular Pathophysiology and Genomics Research Unit, School of Physiology, Faculty of Health Sciences, University of the Witwatersrand, Johannesburg 2000, South Africa

**Keywords:** malondialdehyde, systemic lupus erythematosus, disease damage, musculoskeletal complications, complement system

## Abstract

Malondialdehyde (MDA) is a marker of oxidative stress and antioxidant status. Oxidative stress has been observed to be increased in systemic lupus erythematosus (SLE). Some studies have shown that MDA is upregulated in SLE compared to controls. However, the literature lacks reports regarding the relationship of MDA to disease manifestations. This is relevant since SLE is a multisystemic disease which may affect virtually any organ in the body. In this study, we set out to analyze how MDA serum levels are associated with disease expression in a large series of SLE patients who were fully characterized in clinical and laboratory terms. A total of 284 patients with SLE were recruited. Serum levels of MDA, and the activity (SLEDAI), severity (Katz) and damage index (SLICC-DI) scores, full lipid profile, and carotid subclinical atherosclerosis were assessed. In addition, a full characterization of the complement system was performed in SLE patients’ samples. Multivariable linear regression analysis was executed to study the relationship between clinical and laboratory disease characteristics and MDA. A statistically significant negative relationship was found between disease duration and MDA. In contrast, the presence of anti-nucleosome antibodies was positively associated with MDA. Regarding the SLICC-DI areas, both the musculoskeletal domain and the cutaneous domain were significantly related to higher serum MDA values. Furthermore, after adjustment for confounding factors, lower levels of the classical complement pathway, which denotes activation, were associated with higher serum levels of MDA. In conclusion, cumulative musculoskeletal and skin damage in SLE patients is associated with superior serum levels of MDA. In addition, activation of the complement system is also related to higher circulating MDA levels.

## 1. Introduction

Protein phosphorylation, activation of various transcriptional factors, apoptosis, immunity, and differentiation depend on a proper oxidation process by biological systems. Mitochondria are intracellular organelles that are the major cellular source of reactive oxygen species—ROS—and they are essential for aerobic metabolism and energy production through oxidative phosphorylation, which is accomplished by the respiratory chain. Oxidative stress occurs when the antioxidant system is overwhelmed by the overproduction of ROS, leading to the oxidation of major cellular macromolecules, and resulting in molecular dysfunction. Therefore, oxidative stress plays a critical role in the development of endothelial dysfunction, cancer, inflammation, and atherogenesis [1]. It occurs when there is an excessive production of free radicals or low levels of antioxidants. Malondialdehyde (MDA) is a final product of the cellular peroxidation of polyunsaturated fatty acids and is widely recognized as a marker of oxidative stress and antioxidant status [2]. In neutral solution, MDA is present as the relatively unreactive enolate anion, or at a lower pH as the β-hydroxyacrolein, and can thus form adducts. Under physiological conditions, MDA reacts primarily with the ε-amino groups of protein lysine residues, and has been observed to cause protein cross-linking. A basal level of lipid peroxidation may contribute to the formation of these adducts, and an elevated level of adducts would be indicative of a pathological condition [3]. MDA has been extensively investigated as a biomarker for lipid peroxidation in several diseases and animal models, primarily due to its straightforward detection method. Typically, MDA levels are measured in different blood compartments (plasma, serum, lymphocytes) and tissues using a colorimetric assay that relies on the reaction between MDA and thiobarbituric acid.

Systemic lupus erythematosus (SLE) is a chronic autoimmune disease characterized by its diverse clinical manifestations, unpredictable flare-ups, and involvement of multiple organs. Patients with SLE can present with a wide range of symptoms, from joint and skin involvement to potentially life-threatening complications affecting the kidneys, blood cells, or central nervous system. Notably, SLE patients face an increased risk of premature atherosclerosis compared to individuals with other inflammatory conditions. In this sense, although patients with SLE and rheumatoid arthritis carry higher odds of premature atherosclerotic cardiovascular disease [4], this risk seems to be higher in SLE [5]. Cardiovascular disease has been identified as a leading cause of mortality among SLE patients [5].

Oxidative stress has been observed to be increased in systemic lupus erythematosus (SLE) [6]. This oxidative stress plays a significant role in the pathogenesis of SLE by contributing to immune system dysregulation and various disease manifestations. In SLE, oxidative stress can lead to abnormal activation and processing of cell-death signals and promotes the release of inflammatory lipid hydroperoxides from dysfunctional mitochondria in T cells [7]. These lipid hydroperoxides are highly diffusible and can spread oxidative stress to other intracellular organelles and through the bloodstream, further exacerbating the damage. Elevated levels of MDA have been associated with many clinical features like lupus nephritis and tissue damage in SLE and its association with cardiovascular disease in SLE patients [8]. One important consequence of oxidative stress in SLE is the oxidative modification of self-antigens [9]. This modification occurs when ROS interact with self-proteins, resulting in structural changes and the generation of neoepitopes [9]. These modified self-antigens can trigger an autoimmune response, leading to the production of autoantibodies, which are a hallmark of SLE. Moreover, the degree of oxidative modification of serum proteins has been found to correlate with disease activity and organ damage in SLE [10].

The literature lacks studies on the role of oxidative stress biomarkers in well-defined series of patients with SLE. Since SLE is a multisystemic disease which may affect virtually any organ in the body, the study of the relationship between disease features and MDA is mandatory. If they were related, we could hypothesize that MDA production may be involved in the pathogenesis of the disease. In this work, we aimed to determine how serum MDA (measured through the thiobarbituric acid reactive substance assay), a marker of lipid peroxidation, is related to disease characteristics, including disease activity, damage, and severity in a fully characterized series of 284 SLE patients. We also evaluated lipid profiles, insulin resistance, and subclinical atherosclerosis, as well as performing a complete characterization of the complement system in patients with SLE and analyzed the relationship of these parameters with MDA.

## 2. Materials and Methods

### 2.1. Study Participants

This cross-sectional study enrolled a total of 284 patients diagnosed with systemic lupus erythematosus (SLE). Patients were selected by consecutive recruitment during 2020 and 2021 from the outpatient clinics of two different hospitals in Spain. The inclusion criteria for the study required patients to be 18 years of age or older, have a clinical diagnosis of SLE, and meet at least four classification criteria for SLE as defined by the American College of Rheumatology/European League Against Rheumatism [11]. The patients were identified by rheumatologists and received regular follow-up care at rheumatology outpatient clinics. Participants who were taking prednisone, at a dosage equivalent to or below 10 mg/day, were approved to participate since glucocorticoids are commonly used in the treatment of SLE. The research was conducted in accordance with the principles of the Declaration of Helsinki and was approved by the Institutional Review Committees at Hospital Universitario de Canarias and Hospital Universitario Doctor Negrín, both located in Spain. All study subjects provided informed written consent prior to their participation (Approval Number 2015_84).

### 2.2. Data Collection

Participants included in the study underwent a comprehensive evaluation that included completing a questionnaire on cardiovascular risk factors and medication use. They also underwent a thorough physical examination conducted under standardized conditions. Measurements such as weight, height, body mass index (BMI), abdominal circumference, and blood pressure (both systolic and diastolic, with the participant in a supine position) were recorded. Information regarding smoking status and hypertension treatment was obtained from the questionnaire, while medical records were reviewed to gather specific diagnoses and medication information. 

The activity of SLE and the extent of damage were assessed using the Systemic Lupus Erythematosus Disease Activity Index-2000 (SLEDAI-2K) [12] and the Systemic Lupus International Collaborating Clinics/American College of Rheumatology Damage Index (SLICC/ACR SDI) [13], respectively. In the present study, the SLEDAI-2K index was categorized into different levels of activity: none (0 points), mild (1–5 points), moderate (6–10 points), high (11–19 points), and very high activity (>20 points), as previously described [14]. The severity of the disease was measured using the Katz index [15]. Carotid ultrasound was performed to assess the thickness of the carotid artery wall (carotid intima-media wall thickness or cIMT) in the common carotid artery. Additionally, the ultrasound was used to identify focal plaques in the extracranial carotid artery, following the consensus definitions outlined in the Mannheim criteria [16].

### 2.3. Complement System and MDA Assessment

The thiobarbituric acid reactive substance (TBARS) assay is a method employed to detect lipid oxidation. This assay specifically measures malondialdehyde (MDA), one of the end products generated during the breakdown of lipid peroxidation compounds. Serum levels of MDA were determined using a modified version of the method described by Kikugaw et al. [17]. To perform the assay, a 0.2 mL volume of the sample was combined with 0.2 mL of 0.2 M H_3_PO_4_ (purity 85%; Merck Life Science, Madrid, Spain). The color reaction was initiated by adding 25 µL of a 0.11 M thiobarbituric acid (TBA, purity 100%; Sigma-Aldrich, Madrid, Spain) solution. The mixture was then heated at 90 °C for 50 min using a heating block. After cooling, the TBARS (resulting in a pink complex color) were extracted by adding 0.4 mL of *n*-butanol (purity 100%; Sigma-Aldrich, Madrid, Spain). Centrifugation at 6000× *g* for 10 min allowed for the separation of the butanolic phase. Each sample was transferred to a 96-well plate and read at 535 nm using a microplate spectrophotometer reader (Spectra MAX-190; Molecular Devices, Sunnyvale, CA, USA). A calibration curve was prepared using authentic MDA standards (purity 100 %; Merck Life Science, Madrid, Spain). The detection limit of the assay was determined to be 0.079 nmol/mL. The intra- and inter-assay coefficients of variation were calculated as 1.82% and 4.01%, respectively. The serum concentration of MDA was expressed in nmol per mL. To minimize potential interferences from compounds that react or absorb at 532 nm, each sample was accompanied by a blank tube (sample without the TBA reagent), and the absorbance of the blank tube was subtracted from each sample measurement [18]. Additionally, the use of butanol as the extracting agent for the TBARS complex helped to mitigate many of these interferences [19].

The SVAR functional complement assays, developed by Wieslab^®^ in Sweden, were employed to evaluate the activity of the classical, alternative, and lectin routes of the complement system. These assays utilize the principles of the hemolytic assay for complement function and incorporate labeled antibodies that specifically target the neoantigen generated as a result of complement activation. The quantity of neoantigen produced is directly proportional to the functional activity of the complement pathways. To perform the assays, microtiter strip wells are coated with pathway-specific activators for the classical, alternative, or lectin pathway. The patient’s serum is then diluted in a blocking diluent containing a specific blocker, ensuring that only the pathway under investigation is activated. During the incubation of the diluted patient serum in the wells, the specific coating triggers complement activation. After washing the wells, C5b-9 (membrane attack complex) is detected using an alkaline phosphatase-labeled specific antibody that binds to the neoantigen expressed during membrane attack complex formation. Subsequently, specific antibodies are detected by incubating with an alkaline phosphatase substrate solution. The intensity of color, measured in terms of absorbance (optical density), corresponds to the amount of complement activation. The level of formed membrane attack complex (neo-epitope) indicates the activity of the complement cascade. The results are expressed semi-quantitatively by calculating the optical density ratio between a positive control and the sample. It is important to note that the classical, alternative, and lectin cascade values should be interpreted in such a way that lower levels indicate greater activation of the respective pathway. Wieslab^®^ has certified these functional assays by studying their correlation and concordance with the classical CH50 and AH50 hemolytic tests (source: https://www.svarlifescience.com/ accessed on 15 March 2023). Turbidimetry (Roche, Vienna, Austria) was employed to analyze C2, C3, C3a, C4, and C1q. Nephelometry (Siemens, Erlangen, Germany) was used to analyze C1-inhibitor, while enzyme-linked immunosorbent assay (ELISA Duoset; R&D, Minneapolis, MN, USA) was utilized for the assessment of factor D and factor H. Both the intra- and inter-coefficients of variation for these assays were less than 10%.

### 2.4. Statistical Analysis

Demographic and clinical characteristics of patients with SLE were presented as mean ± standard deviation (SD) or percentages for categorical variables. For continuous variables that did not follow a normal distribution, data were reported as median and interquartile range (IQR). To examine the relationship between disease characteristics and circulating MDA levels, multivariable linear regression analysis was conducted. Confounders from demographic and disease-related data were included in the analysis if they exhibited a significant relationship (*p* < 0.20) with both the independent and dependent variables. All statistical analyses were performed using Stata software, version 17/SE (StataCorp, College Station, TX, USA), with a two-sided significance level of 5%. Statistical significance was considered at *p*-values < 0.05.

## 3. Results

### 3.1. Demographics and Disease-Related Data of Patients with Systemic Lupus Erythematosus 

Circulating MDA in patients with SLE had a median of 1.18 nmol/mL (IQR 0.85–1.52) (Table 1). A total of 284 patients were included in the study, with the majority being women (92%) and with an average age of 50 ± 12 years. Body mass index was 28 ± 6 kg/m^2^, and the abdominal circumference was 92 ± 14 cm. Among the classic cardiovascular risk factors, 24% of patients were current smokers, 39% had hypertension, and 30% were obese. Additionally, a quarter of patients were taking statins, and 29% were taking aspirin (Table 1).

The median duration of disease in SLE patients was 16 years (IQR 7–24). The majority of patients had either no disease activity (40%) or mild to moderate activity (39%) based on the SLEDAI score. The SLICC-SDI and Katz indices were 1 (IQR 0–2) and 2 (IQR 1–4), respectively. A SLICC-SDI score of 1 or higher was found in 68% of patients. Prednisone was being used by 50% of patients, with a median daily dose of 5 mg/day (IQR 5–7.5). At the time the study was performed, 67% of patients tested positive for anti-DNA antibodies, and 69% for anti-ENA antibodies, with anti-SSA being the most commonly detected antibody (35%). Hydroxychloroquine was being used by 69% of the patients. Other less frequently used antirheumatic drugs included methotrexate (11%) and azathioprine (15%). In terms of carotid atherosclerosis, the mean cIMT was 628 ± 109 microns, and 36% of patients had carotid plaque according to ultrasound assessment. Further information on SLE-related data, lipid profile, and insulin resistance indices can be found in Table 1.

### 3.2. Demographic and Disease Characteristics in Relation to Serum MDA Levels

In the univariable analysis, most disease characteristics, cardiovascular comorbidity, serum lipid molecules, subclinical atherosclerosis, and disease-related data were not associated with serum levels of MDA (Table 2). Only disease duration that had a significant negative relationship and the presence of anti-nucleosome antibodies that was positively associated were disease-related data that revealed significant relationships with MDA. Remarkably, SLICC-SDI, SLEDAI, and Katz indices did not show association with circulating MDA.

### 3.3. Relationship of Activity Score and Damage and Disease Severity Indices with MDA

Since the activity score and the damage and disease severity indices are a sum of different aspects of SLE, w in Table 3 we show the relationship of each item of these scores with MDA. Regarding the Katz index, no associations were found between the items of this score with MDA. 

Similarly, SLEDAI score items were in general not associated with circulating MDA. In this regard, only the presence of pleurisy (pleuritic chest pain with pleural rub/effusion or pleural thickening), which was present in three patients (1%), was the item that showed a significant relationship with higher levels of circulating MDA. With respect to SLICC-SDI areas, both the musculoskeletal (*n* = 89) and the skin domains (*n* = 39) were significantly related to higher values of serum MDA (Table 3).

A full list of individualized SLICC-SDI items is shown in Supplementary Table S1. In this representation of all the SLICC items, myocardial infarction ever (*n* = 2, 1%) and skin extensive scarring or panniculus (*n* = 10, 4%) were the individual items that were significantly and positively associated with MDA (Supplementary Table S1).

### 3.4. Multivariable Analysis of the Relationship of Complement System Pathways and Components to MDA

**Figure 1:**
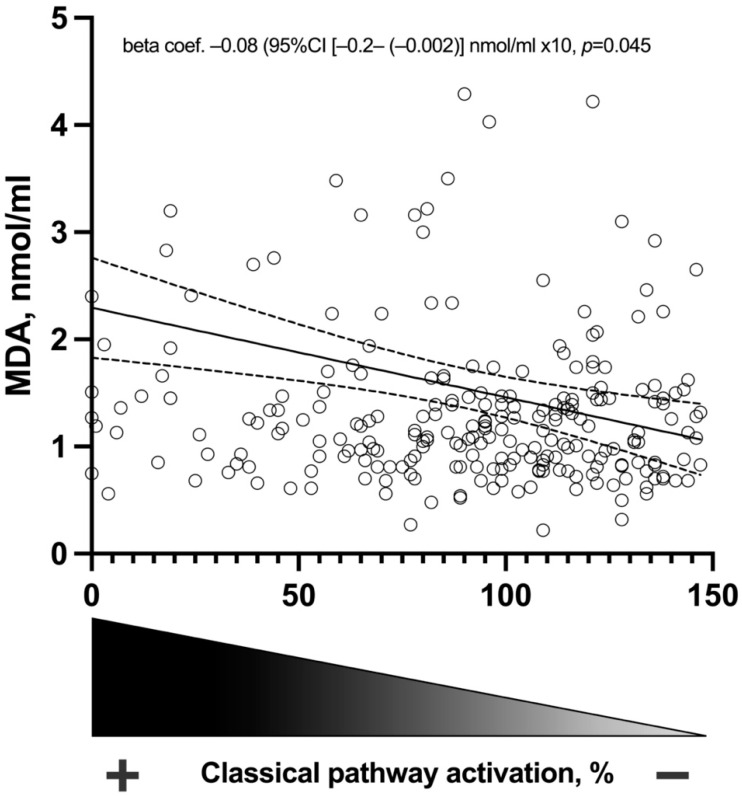
Relation of MDA serum levels to complement classical functional pathway assay.

Serum values of C1q, C2, C3, C3a, factors H and D, C1-inhibitor, as well as functional assays of the three classical, alternative, and lectin pathways are shown in Table 4. In the univariable analysis, the functional assay of the classical pathway revealed a significant relationship with lower circulating MDA (beta coefficient −0.08 (95% confidence interval [−0.1–−0.04] nmol/mL × 10, *p* = 0.001). This relationship was found to be negative, meaning therefore that lower levels of the classical pathway, which denote activation, were associated with higher serum levels of MDA. Remarkably, after adjustment for confounder factors, this relationship remained significant (beta coefficient −0.08 (95% confidence interval [−0.2–−0.002] nmol/mL × 10, *p* = 0.045) (Table 4 and Figure 1).

## 4. Discussion

Our study represents the largest series of patients with SLE in which serum MDA levels and their association with disease expression have been investigated. According to our results, MDA levels were found to be associated with musculoskeletal and cutaneous manifestations of the disease and related to complement activation and consumption after multivariable analysis. Our work emphasizes the potential role that oxidative stress could play in the pathogenesis of certain features of SLE.

Oxidative stress and the presence of free radicals are widely recognized as harmful to human health. Numerous studies have shown that free radicals can cause impairment to cells and tissues, which ultimately contribute to the development and progression of various diseases, including cardiovascular disease and cancer [20]. Due to this, in recent years, various antioxidants such as vitamin E, flavonoids, and polyphenols have been exploited, with some clinical success, due to their real or supposed beneficial effect against oxidative stress. For this reason, the study of the phenomenon of oxidative stress in patients with SLE is of potential interest given the multisystemic expression of the disease and the relatively high percentage of patients who fail with existing therapies. 

Oxidative stress is a common occurrence in autoimmune diseases, characterized by the excessive production of reactive oxygen species (ROS) and reactive nitrogen species. This imbalance in the production of these molecules plays a significant role in the pathogenesis of autoimmune diseases [21,22]. For example, oxidative stress is known to be a dynamic and complex phenomenon occurring in rheumatoid arthritis and it seems to be implicated in the disease pathogenesis [23]. However, to date, a limited number of studies have demonstrated the potential beneficial effects of antioxidant therapies on clinical and biochemical parameters in people with rheumatoid arthritis [24]. These studies offer insight into the possibility of using antioxidant therapies to alleviate disease-related damage, in combination with conventional therapies. This may also be the case for SLE. Mice deficient in NADPH oxidases acquire evidently exacerbated lupus, suggesting that the lack of normal NOX2-dependent cell death may lead to the pathogenesis of this autoimmune disease [25]. In addition, measures of oxidative stress can be associated with disease activity in both cross-sectional and prospective studies of SLE patients [26]. Similarly, higher nitric oxide levels are linked with renal damage and a lack of response to therapy [27,28]. 

Elevated plasma MDA representing oxidative stress have been reported in SLE patients with different clinical implications. For instance, in a study that involved 80 SLE patients and 80 healthy controls, circulating MDA was found to be higher in patients with SLE [29]. MDA also correlated to SLEDAI, and was found to be higher in patients with neuropsychiatric manifestations, vasculitis, and anti-DNA antibodies. However, the comparison of MDA levels between patients with and without disease damage did not yield significant differences [29]. Similarly, in a report of 40 SLE patients and 50 controls, MDA was found to be significantly elevated [30]. MDA has also been described to be associated with alopecia and nephritis in SLE [31]. Other reports have focused on MDA-modified proteins and their relationship with disease features. For example, MDA-modified LDL cholesterol has been linked to the appearance of premature cardiovascular disease and probably also to other autoimmune phenomena seen in SLE [32]. However, in our study we did not find a relationship between circulating MDA and the indices of activity, severity, or damage of the disease. We think this may be because of the fact that the majority of our patients had low or inactive activity levels. However, when the clinical domains of these indices were analyzed separately, a positive relationship was found between cutaneous and joint manifestations with MDA. On the other hand, the immunity profile, except for anti-nucleosome antibodies, did not show a relationship with serum levels of MDA. This was also the case for the lipid pattern, insulin resistance indices, and the presence of subclinical atherosclerosis, which showed no relationship with MDA. Among them, the absence of a relationship between MDA and the lipid profile is remarkable. It is possible that the presence of inflammatory dyslipidemia in patients with SLE is not associated to the peroxidation of molecules related to lipids, or that MDA levels are not responsible for the abnormal lipid profile that patients with SLE present. In this sense, it is important to note that the number of patients with SLE recruited in our study was higher than in the previously mentioned studies. This large number of patients allowed us to perform multivariable analysis.

Pleurisy, but not pericarditis, was associated with MDA. However, the number of patients with these manifestations was low (three patients with pleurisy and one with pericarditis). The low number of subjects with these manifestations prevents drawing a clear conclusion about these associations. In addition, MDA was related to features considered mild but not to others that implied a higher damage. We also do not have a clear explanation for this. MDA may be more related to these joint and skin manifestations and not to others due to hitherto unknown pathological mechanisms.

In SLE, the classical pathway serves as the primary pathway for complement activation. This activation is initiated by the binding of C1q to immune complexes, playing a crucial role in the pathogenesis of the disease [33]. In our work, we found that complement classical cascade activation correlated with MDA serum levels. This was found after multivariable analysis. Complement activation following oxidative stress has been described previously in the literature. In this sense, ROS, such as hydrogen peroxide, have been shown to directly activate C5 via a nonenzymatic mechanism, and inhibition of ROS formation decreases complement activation and deposition [34]. In a previous report in 16 patients with SLE, MDA increased in the low AH50 (alternative complement pathway assay) compared to the normal AH50 patients [35]. However, in this report, other complement routes assays were not assessed, and it lacked multivariable analysis. We believe that, probably, oxidative stress in SLE may be a consequence of the tissular damage produced in the disease and that, subsequently, will generate complement consumption. Our finding on the association between complement consumption/activation and MDA reinforces the affirmation of a pathophysiological relationship between both processes, MDA and the complement system, in patients with SLE.

We acknowledge several limitations in this study. First, although MDA is a substance resulting from oxidative stress, this process is complex and there are other markers associated with this phenomenon that have not been measured in our work. For example, superoxide dismutase catalyzes the dismutation of superoxide into oxygen and hydrogen peroxide. Because of this, this enzyme is known as an important antioxidant defense in most cells exposed to oxygen radicals. Second, similarly, glutathione peroxidase catalyzes the oxidation reaction in reduced glutathione to oxidized glutathione using hydrogen peroxide. The main function of glutathione peroxidase is, therefore, to protect the organism from the degrading effect of hydroperoxides endogenously. Further studies regarding the role of these enzymes in SLE are warranted. Also, the cross-sectional design of our study precludes inferring causality. Third, we have not recruited controls but the presence of a state of oxidative stress compared to controls has already been demonstrated elsewhere [9]. Fourth, thirty percent of the patients were not taking antimalarials. This percentage can be considered high since the use of these is recommended in most cases in patients with SLE [36]. However, the intake of these drugs did not show a relationship with MDA levels. For this reason, we believe that the fact that almost a third of the patients did not take antimalarials has not affected our results. 

## 5. Conclusions

Overall, oxidative stress in SLE may contribute to immune dysregulation, abnormal cell signaling, autoantibody production, and the development of comorbidities. Understanding and targeting oxidative stress pathways may hold promise for the development of new therapeutic strategies in the management of SLE. Besides, we believe assessing MDA serum levels in SLE may contribute to the identification of several disease manifestations and help in the management of the disease.

## Figures and Tables

**Table 1:** Characteristics of SLE patients.

	SLE Patients
	(*n* = 284)
MDA, nmol/mL	1.18 (0.85–1.52)
Age, years	50 ± 12
Female, *n* (%)	261 (92)
Body mass index, kg/m^2^	28 ± 6
Abdominal circumference, cm	93 ± 14
Hip circumference, cm	103 ± 12
Waist-to-hip ratio	0.90 ± 0.07
Systolic pressure, mmHg	127 ± 20
Diastolic pressure, mmHg	79 ± 11
Cardiovascular co-morbidity	
Smoking, *n* (%)	69 (24)
Diabetes, *n* (%)	16 (6)
Aspirin, *n* (%)	80 (29)
Statins, *n* (%)	72 (25)
Obesity, *n* (%)	85 (30)
Hypertension, *n* (%)	111 (39)
Lipid profile	
LDL cholesterol, mg/dL	111 ± 29
HDL cholesterol, mg/dL	61 ± 19
LDL:HDL cholesterol ratio	1.96 ± 0.75
Non-HDL cholesterol, mg/dL	137 ± 33
Cholesterol, mg/dL	198 ± 36
Triglycerides, mg/dL	130 ± 78
Apolipoprotein A1, mg/dL	173 ± 35
Apolipoprotein B, mg/dL	95 ± 23
Apo B: Apo A1 ratio	0.57 ± 0.17
Atherogenic index	3.5 ± 1.1
Lipoprotein (a), mg/dL	39 (12–108)
Carotid intima media thickness, microns	628 ± 109
Carotid plaque, *n* (%)	99 (36)
SLE-related data	
Disease duration, years	16 (7–24)
CRP, mg/dL	2.0 (0.8–4.4)
SLICC-DI	1 (0–2)
SLICC-DI ≥ 1, *n* (%)	191 (68)
Katz Index	2 (1–4)
Katz ≥ 3, *n* (%)	126 (44)
SLEDAI-2K	2 (0–4)
SLEDAI-2K categories, *n* (%)	
No activity, *n* (%)	109 (40)
Mild, *n* (%)	107 (39)
Moderate, *n* (%)	41 (15)
High, *n* (%)	10 (4)
Very High, *n* (%)	4 (1)
Auto-antibodies profile	
Anti-DNA positive, *n* (%)	151 (67)
Anti-ENA positive, *n* (%)	164 (69)
Anti-RNP, *n* (%)	64 (28)
Anti-Sm, *n* (%)	24 (10)
Anti-ribosome	13 (9)
Anti-nucleosome	32 (22)
Anti-histone	22 (15)
Anti-SSA, *n* (%)	55 (35)
Anti-SSB, *n* (%)	36 (21)
Antiphospholipid syndrome, *n* (%)	43 (16)
Antiphospholipid autoantibodies, *n* (%)	61 (32)
Anti beta2 glycoprotein IgM, *n* (%)	19 (10)
Anti beta2 glycoprotein IgG, *n* (%)	28 (15)
Lupus anticoagulant, *n* (%)	51 (28)
ACA IgM, *n* (%)	22 (11)
ACA IgG, *n* (%)	39 (20)
Current prednisone, *n* (%)	140 (50)
Prednisone, mg/day	5 (5–7.5)
Hydroxychloroquine, *n* (%)	194 (69)
Azathioprine, *n* (%)	43 (15)
Methotrexate, *n* (%)	31 (11)
Mycophenolate mofetil, *n* (%)	31 (11)
Rituximab, *n* (%)	8 (3)
Belimumab, *n* (%)	8 (3)

Data are presented as mean ± SD or median (interquartile range) when data were not normally distributed. SLEDAI-2K: Systemic Lupus Erythematosus Disease Activity Index. SLEDAI-2K categories were defined as: 0, no activity; 1–5 mild; 6–10 moderate; >10 high activity, >20 very high activity. SLICC-DI: Systemic Lupus International Collaborating Clinics/American College of Rheumatology Damage Index; MDA: Malondialdehyde; CRP: C reactive protein; LDL: low-density lipoprotein. HDL: high-density lipoprotein; Anti-ENA: extractible nuclear antibodies; ACA: anticardiolipin.

**Table 2:** Demographics and disease features in relation to MDA serum levels.

	MDA, nmol/mL	
	Beta Coef. (95%), *p*	
Age, years	0.007 (−0.009–0.02)	0.38
Female	−0.3 (−1–0.4)	0.35
Body mass index, kg/m^2^	0.02 (−0.02–0.05)	0.35
Abdominal circumference, cm	0.005 (−0.008–0.02)	0.43
Hip circumference, cm	0.009 (−0.006–0.009)	0.24
Waist-to-hip ratio	−0.2 (−3–2)	0.85
Systolic pressure, mmHg	0.001 (−0.008–0.01)	0.75
Diastolic pressure, mmHg	0.006 (−0.01–0.02)	0.44
Cardiovascular co-morbidity		
Smoking	−0.07 (−0.5–0.4)	0.75
Diabetes	0.2 (−0.6–1)	0.66
Hypertension	0.01 (−0.4–0.4)	0.94
Obesity	0.2 (−0.2–0.6)	0.41
Statins	0.03 (−0.4–0.5)	0.87
Aspirin	−0.1 (−0.5–0.3)	0.52
Lipid profile		
Cholesterol, mg/dL	−0.003 (−0.009–0.002)	0.19
Triglycerides, mg/dL	−0.001 (−0.003–0.001)	0.42
LDL cholesterol, mg/dL	−0.005 (−0.01–0.002)	0.15
HDL cholesterol, mg/dL	0.002 (−0.008–0.01)	0.75
LDL: HDL cholesterol ratio	−0.2 (−0.5–0.05)	0.11
Non-HDL cholesterol, mg/dL	−0.005 (−0.01–0.0008)	0.10
Lipoprotein (a), mg/dL	0.0002 (−0.002–0.002)	0.88
Apolipoprotein A1, mg/dL	0.002 (−0.004–0.007)	0.56
Apolipoprotein B, mg/dL	−0.003 (−0.01–0.005)	0.46
Apo B:Apo A1 ratio	−0.6 (−1.7–0.5)	0.27
Atherogenic index	−0.1 (−0.3–0.05)	0.16
cIMT, microns	0.002 (−0.0005–0.003)	0.15
Carotid plaque	0.3 (−0.1–0.7)	0.14
SLE-related data		
Disease duration, years	**−0.02 (−0.04−(−0.005))**	**0.013**
CRP, mg/dL	−0.002 (−0.02–0.01)	0.78
SLICC-DI	0.03 (−0.07–0.1)	0.55
SLICC-DI ≥ 1	0.3 (−0.08–0.7)	0.12
Katz Index	−0.05 (−0.1–0.04)	0.32
Katz ≥ 3	−0.3 (−0.7–0.08)	0.12
SLEDAI	−0.05 (−0.1–0.0003)	0.051
SLEDAI categories		
No activity	ref.	
Mild	−0.08 (−0.5–0.3)	0.72
Moderate to very high	−0.04 (−1–0.08)	0.098
Auto-antibody profile		
Anti-DNA positive	0.2 (−0.2–0.5)	0.44
Anti-ENA positive	0.3 (−0.2–0.7)	0.25
Anti-RNP	−0.2 (−0.7–0.2)	0.30
Anti-Sm	−0.04 (−0.7–0.6)	0.91
Anti-ribosome	−0.4 (−2–0.6)	0.42
Anti-nucleosome	**1 (0.3–2)**	**0.005**
Anti-histone	−0.4 (−1–0.3)	0.27
Anti-SSA	−0.08 (−0.6–0.5)	0.79
Anti-SSB	−0.6 (−2–0.6)	0.30
Antiphospholipid syndrome	−0.5 (−1–0.07)	0.085
Antiphospholipid autoantibodies		
Lupus anticoagulant	−0.04 (−0.6–0.6)	0.89
Anti beta2 glycoprotein IgM	−0.3 (−1–0.6)	0.54
Anti beta2 glycoprotein IgG	−0.6 (−1–0.1)	0.099
ACA IgM	−0.2 (−1–0.6)	0.66
ACA IgG	−0.5 (−1–0.2)	0.15
Current prednisone	−0.3 (−0.7–0.07)	0.11
Prednisone, mg/day	0.03 (−0.02–0.08)	0.30
Hydroxychloroquine	0.2 (−3–3)	0.91
Methotrexate	0.3 (−0.3–0.9)	0.30
Mycophenolate mofetil	−0.04 (−0.6–0.5)	0.88
Azathioprine	−0.4 (−0.9–0.08)	0.10
Rituximab	−0.3 (−2–0.9)	0.59
Belimumab	−0.6 (−1.8–0.6)	0.31

In this analysis, MDA is considered the dependent variable. MDA: Malondialdehyde. Significant *p* values are depicted in bold; SLEDAI-2K: Systemic Lupus Erythematosus Disease Activity Index. SLEDAI categories were defined as: 0, no activity; 1–5 mild; 6–10 moderate; >10 high activity, >20 very high activity; SLICC-DI: Systemic Lupus International Collaborating Clinics/American College of Rheumatology Damage Index; HDL: high-density lipoprotein; Anti-ENA: extractible nuclear antibodies; ACA: anticardiolipin; cIMT: carotid intima thickness; CRP: C reactive protein; LDL: low-density lipoprotein.

**Table 3:** Individual disease score items in relation to MDA serum levels.

			MDA, nmol/mL	
	*n*	%	Beta Coef. (95%)	*p*
Katz index				
History of cerebritis (seizure or organic brain syndrome)	12	6	−0.1 (−1–0.3)	0.56
History of pulmonary disease	10	5	−0.2 (−1–0.3)	0.47
Biopsy proven diffuse proliferative glomerulonephritis	23	12	−0.03 (−0.3–0.2)	0.86
4–6 ARA criteria for SLE satisfied to date	139	73	0.5 (−0.004–1)	0.052
7 or more ARA criteria for SLE satisfied to date	23	12	−0.2 (−1–0.1)	0.23
History of proteinuria (2+ or more)	62	32	−0.3 (−1–0.2)	0.23
Lowest recorded hematocrit to date = 30–37%	88	46	0.01 (−0.4–0.4)	0.97
Lowest recorded hematocrit to date < 30%	47	25	−0.06 (−0.3–0.2)	0.67
Highest recorded creatinine to date = 1.3–3	28	15	−0.08 (−0.7–0.5)	0.80
Highest recorded creatinine to date > 3	3	2	0.02 (−1–1)	0.96
SLEDAI-2K				
Seizures	1	0	0.4 (−3–3)	0.79
Psychosis	1	0	−1 (−4–2)	0.55
Organic brain syndrome	0	0	−	
Visual disturbance	1	0	−	
Cranial nerve disorder	1	0	−	
Lupus headache	1	0	−0.3 (−3–3)	0.85
ACVA	0	0	−	
Vasculitis	1	0	−0.2 (−3–3)	0.88
Arthritis	9	3	−0.5 (−2–1)	0.40
Myositis	0	0	−	
Urinary cylinders	7	3	−0.3 (−2–1)	0.71
Hematuria	16	6	−0.4 (−1–1)	0.45
Proteinuria	5	2	−0.3 (−2–1)	0.65
Pyuria	11	4	−0.4 (−1–1)	0.39
Rash	21	8	−0.3 (−1–0,4)	0.38
Alopecia	11	4	−0.01 (−1–1)	0.98
Mucosal ulcers	14	5	0.05 (−1–1)	0.91
Pleurisy	3	1	**4 (2–6)**	**<0.001**
Pericarditis	1	0	1 (−2–4)	0.56
Low complement	76	28	0.2 (−0,2–1)	0.29
Elevated anti-DNA	85	31	−0.2 (−1–0,2)	0.44
Fever	2	1	−0.4 (−3–3)	0.81
Thrombopenia	10	4	−0.4 (−1–1)	0.47
Leukopenia	19	7	0.01 (−1–1)	0.98
SLICC-DI domains				
Ocular	63	22	−0.0009 (−0.4–0.4)	0.10
Neuropsychiatric	40	14	−0.3 (−0.9–0.2)	0.21
Renal	28	10	−0.1 (−0.7–0.5)	0.66
Pulmonary	19	7	0.04 (−0.7–0.8)	0.92
Cardiovascular	23	8	0.2 (−0.5–0.8)	0.63
Peripheral vascular	34	12	0.1 (−0.5–0.7)	0.67
Gastrointestinal	28	10	−0.3 (−0.9–0.4)	0.40
Musculoskeletal	89	31	**0.4 (−0.02–0.8)**	**0.041**
Skin	39	14	**0.6 (0.05–1)**	**0.032**
Premature gonadal failure	19	7	−0.1 (−0.8–0.6)	0.77
Diabetes (regardless of treatment)	18	6	0.6 (−0.2–1)	0.14
Malignancy (excluded dysplasia)	11	4	0.08 (−0.9–1)	0.87

The presence of a SLICC domain involvement is shown if points in the domain are ≥ 1. See Supplementary Table S1. ARA: American Rheumatism Association; ACVA: Acute Cerebrovascular Accident; SLICC-DI: Systemic Lupus International Collaborating Clinics/American College of Rheumatology Damage Index. History of pulmonary disease refers to the presence of lupus pneumonitis, pulmonary hemorrhage, or pulmonary hypertension; MDA: Malondialdehyde. Significant *p* values are depicted in bold; SLEDAI: Systemic Lupus Erythematosus Disease Activity Index; SLE: Systemic Lupus Erythematosus. Significant *p* values are depicted in bold.

**Table 4:** Multivariable analysis of the relationship of complement system pathways and components to MDA.

		MDA ×10, nmol/mL			
		Beta Coef. (95%CI), *p*			
		Univariable		Multivariable	
Classical pathway					
Functional assay, %	91 ± 38	**−0.08 (−0.1–(−0.04))**	**0.001**	**−0.08 (−0.2–(−0.002))**	**0.045**
C1q, mg/dL	34 ± 11	0.07 (−0.1–0.2)	0.42		
Lectin pathway					
Functional assay, %	10 (1–41)	−0.006 (−0.05–0.04)	0.79		
Common elements of the classical and lectin pathways					
C2, mg/dL	2.5 ± 1.2	0.4 (−1–2)	0.60		
C4, mg/dL	21 ± 12	−0.07 (−0.2–0.09)	0.40		
C1 inhibitor, mg/dL	32 ± 9	−0.1 (−0.2–0.2)	0.90		
Alternative pathway					
Functional assay, %	41 (12–79)	0.02 (−0.03–0.07)	0.50		
Factor D, ng/mL	2593 ± 1836	−0.00008 (−0.0008–0.0006)	0.83		
Common elements of the three pathways					
C3, mg/dL	130 ± 40	−0.02 (−0.03–0.07)	0.45		
C3a, mg/dL	39 ± 10	−0.05 (−0.2–0.1)	0.62		
Factor H, ng/mL × 10^−3^	389 (281–564)	0.0001 (−0.001–0.002)	0.87		

Data represent mean ± SD or median (interquartile range) when data were not normally distributed. Complement routes and elements are considered the independent variable. Multivariable analysis is adjusted for disease duration and the presence of antiphospholipid syndrome and anti-nucleosome antibodies. Significant *p* values are depicted in bold.

## Data Availability

The data sets used and/or analyzed in the present study are available from the corresponding author upon request.

## Supplementary Materials

The following supporting information can be downloaded at: https://www.mdpi.com/article/10.3390/antiox12081535/s1, Table S1: Relation of SLICC score items to MDA.
