# Supplementary material for: Relationship between Malondialdehyde Serum Levels and Disease Features in a Full Characterized Series of 284 Patients with Systemic Lupus Erythematosus
Source: Antioxidants (Basel). 2023 Jul 31;12(8):1535. doi: 10.3390/antiox12081535 (PMC10451961; doi:10.3390/antiox12081535)
Supplement: Supplementary file 1 [file antioxidants-12-01535-s001.zip › antioxidants-2500493-supplementary.pdf]

Supplementary Table S1. Relation of SLICC-DI score items to MDA

	n	%	MDA, nmol/ml beta coef. (95%)	p
Ocular				
Any cataract ever	29	11	-0.3 (-0.9-0.3)	0.28
Retinal change or optic atrophy	33	12	0.1 (-0.5-0.7)	0.68
Points =>1 in the domain	63	22	-0.0009 (-0.4-0.4)	0.10
Neuropsychiatric				
Cognitive impairment	7	3	0.3 (-1-2)	0.65
Seizures requiring therapy for 6 months	15	5	-0.5 (-1-0.3)	0.26
Cerebrovascular accident ever	13	5	-0.4 (-1-0.3)	0.28
Cranial or peripheral neuropathy	5	2	-0.8 (-2-0.5)	0.24
Transverse myelitis	1	0	-0.5 (-3-2)	0.75
Points =>1 in the domain	40	14	-0.3 (-0.9-0.2)	0.21
Renal				
Estimated or measured glomerular filtration rate <50%	13	5	-0.3 (-1-0.5)	0.43
Proteinuria 3.5 gm/24 hours	7	3	-0.2 (-1-1)	0.75
End-stage renal disease	4	1	0.06 (-0.4-0.6)	0.81
Points =>1 in the domain	28	10	-0.1 (-0.7-0.5)	0.66
Pulmonary				
Pulmonary hypertension	4	1	-0.4 (-2-1)	0.58
Pulmonary fibrosis	4	1	-0.2 (-2-2)	0.88
Shrinking lung	2	1	-0.6 (-3-2)	0.60
Pleural fibrosis	1	0	2 (-1-5)	0.18
Pulmonary infarction	1	0	-0.1 (-3-3)	0.94
Points =>1 in the domain	19	7	0.04 (-0.7-0.8)	0.92
Cardiovascular				
Angina or coronary artery bypass	4	1	1 (-0.3-3)	0.11
Myocardial infarction ever	2	1	**3 (1-5)**	**0.003**
Cardiomyopathy	2	1	-0.7 (-4-2)	0.63
Valvular disease	9	3	0.3 (-0.7-1)	0.58
Pericarditis for 6 months, or pericardiectomy	2	1	0.2 (-3-3)	0.89
Points =>1 in the domain	23	8	0.2 (-0.5-0.8)	0.63
Peripheral vascular				
Claudication for 6 months	3	1	**3 (0.5-5)**	**0.016**
Minor tissue loss (pulp space)	5	2	-0.2 (-2-1)	0.79
Significant tissue loss ever	0	0	-	-
Venous thrombosis	14	5	0.06 (-1-1)	0.91
Points =>1 in the domain	34	12	0.1 (-0.5-0.7)	0.67
Gastrointestinal				
Infarction or resection of bowel	22	8	-0.3 (-1-0.4)	0.34
Mesenteric insufficiency	1	0	-	-
Chronic peritonitis	1	0	-1 (-4-2)	0.49
Stricture or upper gastrointestinal tract surgery ever	0	0	-	-
Pancreatitic insufficiency	0	0	-	-
Points =>1 in the domain	28	10	-0.3 (-0.9-0.4)	0.40
Musculoskeletal				
Muscle atrophy or weakness	3	1	-0.3 (-2-1)	0.66
Deforming or erosive arthritis	40	15	0.3 (-0.2-0.7)	0.25
Osteoporosis with fracture or vertebral collapse	23	9	0.5 (-0.1-1)	0.13

Avascular necrosis	7	3	-0.3 (-1-0.7)	0.57
Osteomyelitis	1	0	-	-
Tendon rupture	4	2	-0.4 (-2-1)	0.57
Points =>1 in the domain	89	31	**0.4 (-0.02-0.8)**	**0.041**
Skin				
Scarring chronic alopecia	16	6	0.04 (-0.7-0.8)	0.92
Extensive scarring or panniculum	10	4	**2 (1-4)**	**<0.001**
Skin ulceration	4	1	0.1 (-2-2)	0.88
Points =>1 in the domain	39	14	**0.6 (0.05-1)**	**0.032**
Premature gonadal failure	19	7	-0.1 (-0.8-0.6)	0.77
Diabetes (regardless of treatment)	18	6	0.6 (-0.2-1)	0.14
Malignancy (exclude dysplasia)	11	4	0.08 (-0.9-1)	0.87

SLICC items and domains represent the independent variable. MDA: Malondialdehyde.
SLICC-SDI: Systemic Lupus International Collaborating Clinics/American College of Rheumatology Damage Index.
Significant p values are depicted in bold.
